# Meldonium Improves Functional Capacity in Patients with Right Ventricular Failure

**DOI:** 10.3390/jcm14217787

**Published:** 2025-11-02

**Authors:** Dana Kigitovica, Krisjanis Dzirnieks, Aivars Lejnieks, Maija Dambrova, Andris Skride, Reinis Vilskersts

**Affiliations:** 1 Riga Stradins University, 16 Dzirciema Str., LV-1007 Riga, Latvia; 2 Pauls Stradins Clinical University Hospital, 13 Pilsonu Str., LV-1012 Riga, Latvia; 3 Riga East Clinical University Hospital, 2 Hipokrata Str., LV-1038 Riga, Latvia; 4 Latvian Institute of Organic Synthesis, 21 Aizkraukles Str., LV-1006 Riga, Latvia

**Keywords:** right ventricular failure, 6MWT, SF-36

## Abstract

**Background/Objectives:** Right ventricular (RV) failure (RVF) is associated with poor prognosis and currently has no known treatment. Meldonium is a clinically used cardiometabolic drug that improves RV function in a preclinical RVF model. This study aimed to assess the safety and efficacy of meldonium in patients with pulmonary arterial hypertension (PAH)-induced RVF. **Methods:** Twenty RVF patients received meldonium (500 mg, b.i.d.) for 30 days; afterward, they were followed up for 30 days. The 6 min walk test (6MWT), 36-Item Short Form Survey (SF-36, a quality-of-life questionnaire), WHO functional class (FC), and Borg dyspnea score (BDS) were used to indirectly assess exercise capacity. Blood samples were obtained before and after treatment and at the end of follow-up. **Results:** Walking distance in the 6MWT increased from 352.2 ± 114.8 m to 398.9.8 ± 128.5 m (*p* = 0.021) after meldonium. Meldonium treatment markedly improved WHO FC and SF-36 scores (*p* < 0.05). The drug significantly improved the BDS after the 6MWT (*p* = 0.003). Meldonium did not affect vital signs or blood biochemistry, including BNP. Meldonium treatment was safe in RVF patients. **Conclusions:** Meldonium treatment increases the functional capacity and overall well-being of RVF patients. Our results suggest that meldonium might be a viable novel drug for RVF treatment.

## 1. Introduction

Right ventricular (RV) failure (RVF) is a clinical syndrome characterized by decreased right ventricular function that leads to suboptimal delivery of blood to the pulmonary circulation and/or elevated venous pressure at rest or during exercise [1,2]. Historically, RVF has received less attention than left ventricular (LV) failure; however, the high mortality rates and poor/decreased quality of life of RVF patients reflect the severity and clinical importance of the syndrome [3,4,5,6]. The main cause of RVF is LV dysfunction–induced pulmonary hypertension (PH); however, RVF can also be induced by RV myocardial infarction, valvular heart diseases and cardiomyopathies [3,4]. Currently, there are no specific drugs for the treatment of RVF [7]. In addition, drugs for the treatment of conditions that induce RVF may attenuate the development of RVF but not improve the function of the right ventricle; specifically, etiological therapy for pulmonary artery hypertension (PAH) targets the pulmonary circulation and decreases blood pressure in pulmonary arteries but has little or no effect on RVF [7,8]. β-Blockers, angiotensin-converting enzyme inhibitors, and mineralocorticoid receptor antagonists have been tested for the treatment of RVF; however, most of them did not improve right ventricular (RV) function [9].

The development of RV failure is characterized by altered myocardial energy metabolism, and modulation of energy metabolism pathways in heart muscle has been suggested as a promising therapeutic option [10]. Both ranolazine and trimetazidine act as metabolic modulators by inhibiting mitochondrial fatty acid oxidation in the myocardium [11]. Clinical study has demonstrated that therapy with trimetazidine, an anti-ischemic (antianginal) metabolic agent, in RVF patients improved 36-Item Short Form Survey (SF-36) quality-of-life scores [12,13]. Another study assessing the efficacy of trimetazidine showed improvement in myocardial velocity on tissue Doppler imaging in both ventricles of patients with heart failure [14]. Therapy with ranolazine, a persistent or late inward sodium current inhibitor and chronic angina treatment, was safe in PAH-induced RVF patients and was associated with an improvement in functional class (FC), a decrease in RV size, and an improvement in RV strain during exercise at 3 months of follow-up [15,16]. In patients with precapillary PH and RV dysfunction, ranolazine improved the RV ejection fraction (RVEF) but had no effect on New York Heart Association (NYHA) class, 6MWT results, or NT-proBNP levels [17]. Thus, improving myocardial energy metabolism is a promising approach to improve RVF patient health status.

Meldonium is a cardiometabolic drug that has shown cardioprotective activity in preclinical models as well as clinical studies [18,19]. The mechanism of action of meldonium is based on the regulation of energy metabolism pathways through an L-carnitine-lowering effect andthe ability of meldonium to redirect long-chain FA metabolism from mitochondria to peroxisomes. Meldonium also stimulates glucose utilization that contributes to the normalization of lipid and glucose metabolic balance [18]. The addition of meldonium to existing treatment in patients with LV heart failure improved their quality of life and FC [18]. Another study showed that better improvement was observed in chronic heart failure (CHF) patients treated concomitantly with meldonium and the angiotensin-converting enzyme inhibitor lisinopril than in those who received angiotensin-converting enzyme inhibitor treatment alone [19]. One study showed that the addition of meldonium in patients with CHF increased the pulmonary arterial flow acceleration time and shortened the RV isovolumic relaxation time and Tei index [20]. Statsenko et al. assessed the effects of combined meldonium and basic therapy in patients with CHF in the early postinfarction period. Clinical improvement and favorable changes in cardiac structural and functional parameters and heart rate variability were noted in the meldonium group [21]. In a recent preclinical study, we showed that meldonium treatment in a monocrotaline-induced rat model of PAH attenuated the development of RV hypertrophy, increased RV fractional area change, decreased the Fulton index and normalized the function of mitochondria in the cardiomyocytes of the right ventricle [22].

In this study, we tested whether meldonium treatment improves the quality of life, physical function, plasma biochemical profile and functional capacity in PAH patients with RVF.

## 2. Materials and Methods

This was an investigator-initiated observational study conducted in P. Stradins Clinical University Hospital, Riga, Latvia, during the period 2021–2022. Consecutive patients in ambulatory practice who met the eligibility criteria were included. Inclusion criteria included (1) Patients ≥ 18 years of age; (2) fulfillment of the criteria for right ventricular failure due to Group 1 PAH and were classified as World Health Organization (WHO) functional class (FC) I to III. Right heart catheterization (RHC) was completed prior to the study in all patients to confirm the diagnosis of PAH according to the 6th World Symposium on Pulmonary Hypertension Task Force criteria: mean pulmonary arterial pressure (mPAP) of ≥20 mmHg and pulmonary capillary wedge pressure (PCWP) ≤ 15 mmHg and pulmonary vascular resistance ≥ 3 Wood units. No other causes of pulmonary hypertension or PAH; (3) belonging to one of the following 2018 Clinical Group 1 subtypes: idiopathic PAH (IPAH), heritable PAH, or PAH associated with connective tissue disease (CTD PAH); (4) a stable treatment regimen with one or more treatments approved for primary disease and CHF. The definition of RVF includes the following parameters: (1) presence of RV systolic dysfunction; (2) signs of right-sided pressure overload; (3) clinical evidence of RVF. Stable therapy was defined as constant therapy for ≥12 weeks before the screening visit and a stable dosage of each medication for ≥8 weeks before the screening visit. Patients remained on their previously prescribed background medications for PH, heart failure and comorbidities without changing the dosage during the study period.

Exclusion criteria included (1) evidence of LV heart failure on the echocardiograms of the enrolled patients (heart failure with reduced ejection fraction); (2) WHO FC IV symptoms; (3) severe or end-stage renal disease and liver failure; and (4) women who were pregnant or lactating.

**Figure 1:**
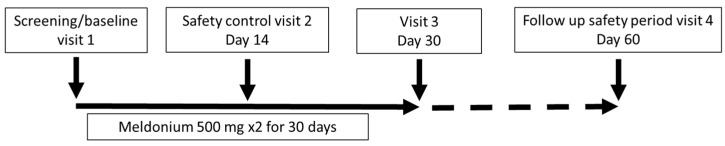
Study schematic.

The study consisted of an initial visit, a safety control visit after 14 days, an examination of the patient after 30 days of treatment ± 7 days, and a health checkup after a washout period of 30 days ± 7 days. The study procedures included a clinical visit with a physical examination; completion of the SF-36; assessment of the Borg dyspnea score (BDS); administration of the 6MWT; laboratory testing, such as full blood count, liver and kidney function tests and B-type natriuretic peptide (BNP); assessment of WHO FC; and determination of adverse events. The 6MWT was assessed in a flat, straight, enclosed corridor that was 30 m long and clearly marked with the start point, a distance marker every 3 m, and the end point. The 6MWT was performed as described in the guidelines [23]. Study participants completed a quality-of-life questionnaire (SF-36) before treatment, just after treatment, and one month after the end of treatment. In addition, at the same time points, all participants underwent a standardized 6MWT, BDS evaluation and assessment of WHO FC; laboratory testing, however, was performed only at visit 3 (Figure 1).

Written informed consent was obtained from all patients before the study. Ethical approval was obtained from the Clinical Research Ethics Committee of the Development Society of Pauls Stradins Clinical University Hospital (Paula Stradiņa klīniskās universitātes slimnīcas Attīstības biedrības Klīniskās izpētes ētikas komiteja, Atzinums Nr. 030221-8L) and the State Agency of Medicines of the Republic of Latvia (16 March 2021) before the initiation of the study. All experiments were performed in accordance with the State Agency of Medicines of the Republic of Latvia. During the initial visit, patients received 60 meldonium (500 mg) capsules, which they were to take orally twice a day for the next 30 days. In the event of a missed dose, patients were instructed to take the next dose as scheduled and not to compensate for the missed dose. The next follow-up visits were scheduled one month and two months after the first visit. During the study, the occurrence of serious adverse effects was assessed. Serious adverse events were defined as a fatal or serious deterioration of health resulting in death, risk of death, hospitalization for >24 h, disability or incapacitation, or intervention to prevent a life-threatening condition.

The primary objectives were to assess the safety and efficacy of treatment with meldonium in patients with RVF. The primary safety endpoints were the incidence of serious adverse events (SAEs) and the incidence of all adverse events (AEs) in the patients. The primary efficacy endpoint was the change in the results of the 6MWT from baseline to the end of treatment at one month.

### Statistical Analysis

Continuous variables were expressed as the mean ± standard deviation. Categorical variables are displayed as counts and percentages. Differences in SF-36 scores, 6 min walk distance, and laboratory parameter assessments were tested using one of two significance tests for continuous variables: the dependent-samples *t* test or the nonparametric Wilcoxon test. A *p* value < 0.05 was considered significant. Statistical analysis was performed using SPSS version 22.0 (IBM Corp., Armonk, NY, USA).

## 3. Results

A total of 22 patients who met the inclusion criteria were enrolled in the study from 2021 to 2022. Two patients refused to continue the study because they were unable to make onsite visits due to the COVID-19 pandemic. During the 2 months of the study, enrolled patients did not report any SAEs or other AEs. The mean age of the patients at the beginning of the study was 70.4 ± 13.2 years, the majority of patients (75%) were female, and PAH was the primary disease—IPAH (*n* = 14) and CTD PAH (*n* = 6). The most common WHO FC at baseline was class III (65%). The demographic and clinical characteristics of the patients, as well as their echocardiography and RHC, are presented in Table 1 and Table A1.

**Figure 2:**
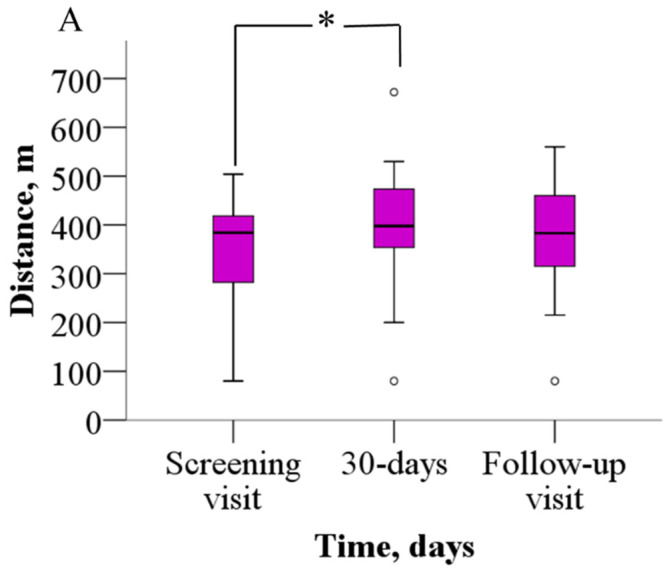

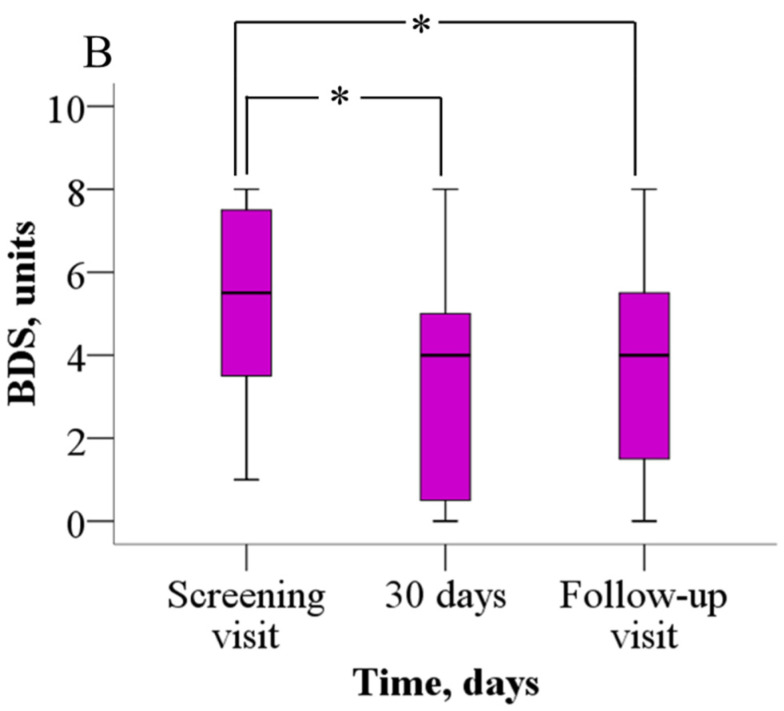
The effect of meldonium on (**A**) 6MWT performance and (**B**) BDS. The graphs represent the increase in 6MWT and decrease in BDS after treatment with meldonium. The results are shown as the mean ± SD of 20 patients; * *p* < 0.05 vs. the value as of the screening visit, paired-sample *t* test for 6MWT, Wilcoxon signed-rank test for BDS. BDS—Borg dyspnea score, 6MWT—six-minute walk test.

The analysis of the 6MWT results revealed that patients were able to walk significantly longer distances after meldonium treatment than before (Figure 2A). Before treatment, patients were able to walk 352.2 ± 114.8 m, but after 30 days of meldonium treatment, the walking distance increased to 398.9 ± 128.5 m (*p* = 0.021), the weighted mean change (wmc) is 46.9 m. On day 60 of the study, the results from the 6MWT demonstrated that the walking distance returned to the pretreatment value (376.7 ± 113.8 m, *p* > 0.05), the wmc is 24.4 m. In addition, treatment with meldonium markedly decreased the BDS (Figure 2B) from the baseline score of 5.4 ± 2.2 to 3.4 ± 2.5 at day 30 (*p* = 0.003), the wmc is −2.1, and the effect persisted at day 60 with a score of 3.7 ± 2.5 (*p* = 0.004), and the wmc is −1.7 (Figure 2B).

**Figure 3:**
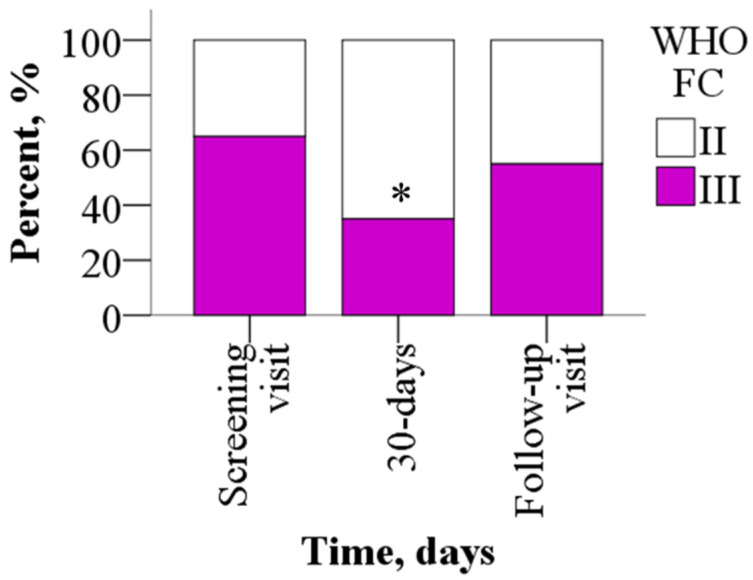
WHO FC before and after treatment with meldonium. Treatment with meldonium improved the functional class of the patients. The results are shown as percentages of 20 patients, * *p* < 0.05 vs. screening visit, chi-square test. WHO FC—World Health Organization functional class.

Sixty-five percent of the study participants reported advanced FC III symptoms at baseline, of whom 65% improved to FC II symptoms (*p* = 0.031) by day 30, while the rest remained WHO FC III. After a 30-day washout period, 55% of the patients were in WHO FC III (*p* > 0.05) (Figure 3).

Heart rate and systemic blood pressure were within the normal range in the majority of patients (Table A2). No significant changes were noticed in the vital parameters after treatment with meldonium, except that there was a marked decrease in diastolic pressure after the 6MWT (*p* = 0.03) at day 30 and a decrease in heart rate after the 6MWT (*p* = 0.04) at day 60.

**Figure 4:**
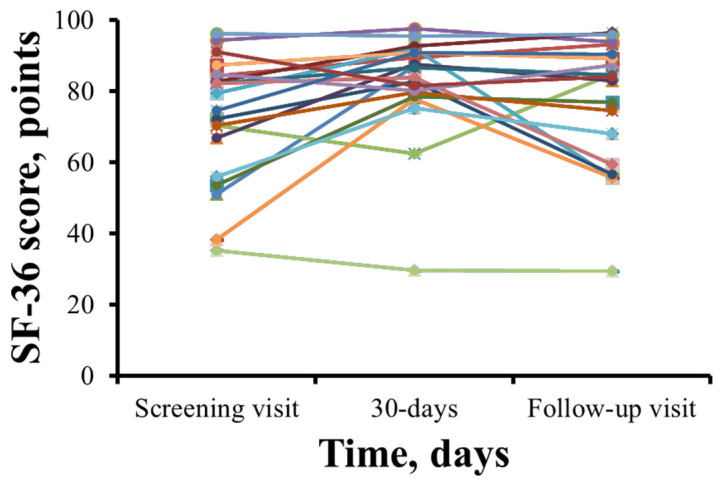
Summary of 36-Item Short Form Health Survey results before and after treatment with meldonium. In most patients, treatment with meldonium improved the SF-36 score at day 30, and a steady decline was noted at the follow-up visit. The results are shown for 20 patients.

The SF-36 health-related quality of life scores for each patient are shown in Figure 4. SF-36 scores in 15 patients increased after 1 month of therapy with meldonium. However, 5 patients reported a decrease in their SF-36 score (Figure 4). The total SF-36 score increased from 72.6 ± 17.7 points during the initial visit to 82.1 ± 14.8 points (*p* = 0.009) after 30 days of meldonium treatment with the wmc of 9.4 points. After the washout period, the SF-36 score decreased to 77.1 ± 17.7 points, and the wmc is 4.5 points.

**Figure 5:**
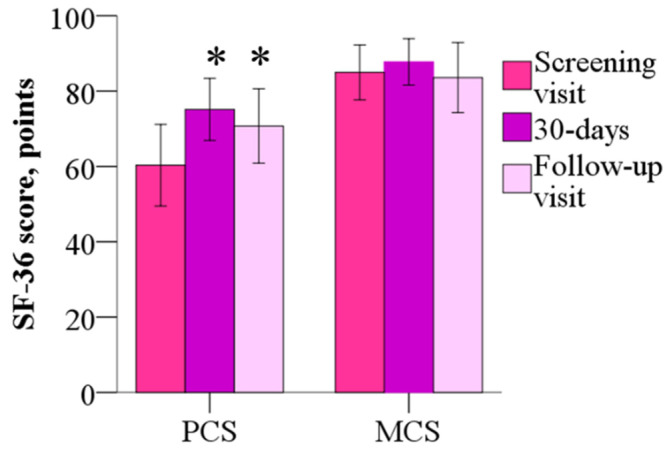
The 36-Item Short Form Health Survey mental component summary and physical component summary before and after 30 days of treatment with meldonium. Treatment with meldonium improved the functional capacity of the patients but had no effect on MCS. The results are shown as the mean of 20 patients; * *p* < 0.05 vs. PCS before the treatment, Wilcoxon signed-rank test. MCS—mental component summary; PCS—physical component summary.

The mean mental component summary (MCS) of the SF-36 was 84.9 ± 15.6 points before the treatment, 87.7 ± 13.2 points (*p* > 0.05) after the 30-day treatment, the wmc of 2.8 points and 83.6 ± 19.8 points at day 60, and the wmc of −1.4 points (*p* > 0.05). As shown in Figure 5, the physical component summary (PCS) score was 60.3 ± 23.1 points before treatment, but after 30 days and 60 days, scores decreased to 75.1 ± 17.6 (the wmc of 14.8 points) and 70.7 ± 21.2 points (the wmc of 10.4 points), respectively (*p* < 0.05). Treatment with meldonium induced improvement in the Physical Functioning, Role–Physical and Bodily Pain domains of the SF-36.

The MCS is composed of role limitations due to emotional problems, social functioning, emotional well-being, and energy/fatigue; none of these subscales showed improved scores after 30 days of treatment with meldonium or after 60 days from the beginning of the study. No changes were noted in the subscore for general health. PCS parameters such as physical functioning and bodily pain improved after 30 days and remained above the screening score at day 60 (*p* < 0.05), but role limitations due to physical health were improved only at day 30 (Table 2).

There were no differences in blood cell counts, liver and kidney functional parameters or BNP levels across the three time points; thus, it can be inferred that these parameters were not influenced by meldonium treatment (Table A3).

## 4. Discussion

This is the first clinical study demonstrating that treatment with meldonium significantly increases daily physical performance and diminishes shortness of breath in patients with chronic RVF due to PAH. Meldonium treatment improved BDS and parameters characterizing objective and subjective physical functioning. At baseline, 65% of patients in this study were in WHO FC III, but after the 30-day meldonium treatment, WHO FC III included only 35% of the patients. In addition, our study demonstrates that meldonium is safe in patients with chronic right heart failure. No major AEs were observed during the 60-day period.

The 6MWT is a commonly used test for the objective assessment of functional exercise capacity for the management of patients with moderate-to-severe pulmonary disease and is especially widely used in patients with PH and RVF [23]. 6MWT performance in PAH patients with RVF can be increased by drugs that alter the function of the pulmonary vasculature. All currently used PAH treatments produce a significant increase in 6MWT performance from baseline to the endpoint of the study [24]. In addition, 24 weeks of treatment with the β-blocker nebivolol was found to increase 6MWT distance, induce a drop in BDS, and lower the FC of the patients [25]. The effects of nebivolol can be attributed to the vasoprotective effects induced by its β2 and β3 agonist properties, as other classical β blockers had no effect on 6MWT distance in RVF patients [26,27,28]. The results from a preclinical PAH-induced RVF model revealed that meldonium treatment had no effect on pulmonary vascular reactivity [22]. Thus, it can be concluded that the improvements induced by meldonium treatment are due to the modification of energy metabolism pathways. The functional status of RVF patients can also be improved by other drugs that modulate energy metabolism. In a study that administered ranolazine to PAH patients for a three-month period, there was an increase in 6MWT performance from 383 ± 60 m to 419 ± 80 m, along with a slight, statistically nonsignificant increase in the Kansas City Cardiomyopathy Questionnaire summary score [16]. Similar findings were reported in a randomized double-blind placebo-controlled trial in PAH patients with trimetazidine: the trimetazidine-treated patients showed an improvement in functional capacity [13]. In our study, meldonium produced remarkable improvements in 6MWT performance (from 352 ± 115 to 399 ± 129 m) and in the physical component summary of the SF-36 after only 1 month of therapy. Moreover, some subscale SF-36 scores and BDS were significantly improved even after a 30-day washout period. This can be explained by the months-long elimination period of meldonium [29,30] and the prolonged presence of meldonium in tissues. In addition, two-thirds of the included patients were in WHO FC III, with marked limitation of physical activity and correspondingly high BNP; nevertheless, meldonium therapy significantly lowered their WHO FC, which demonstrates that meldonium treatment improves conditions after a relatively short treatment period even in patients with severe disease.

The majority of studied patients continued to use more than one drug during the study and had various comorbidities. Meldonium treatment was started as an additional therapy; nevertheless, it induced significant improvements on the background of other drugs and comorbidities without producing significant AEs during the treatment or follow-up period; thus, meldonium can be safely combined with other drugs used by RVF patients, and its efficacy may not be influenced by comorbidities. Hypothetically, the effects of meldonium may not be observed in patients who are already using metabolic modulators, as their energy metabolism has already changed; however, none of the participants in this study reported the use of any other metabolic modulator.

Our observational study shows that meldonium increases exercise capacity in patients with RVF. However, from the existing results, it is not possible to indicate whether the effects of meldonium were due to modified energy metabolism in the myocardium, the skeletal muscles, or both. The increase in 6MWT performance was not accompanied by tachycardia or blood pressure elevation, which indirectly demonstrates an increase in physical tolerance, as well as stable cardiac output. A previous clinical study showed that 12 months of meldonium therapy in patients with stable angina pectoris increased total exercise time and time to the onset of angina, which may indicate modified energy metabolism in the heart muscle [31]. On the other hand, another study showed improvement of exercise tolerance with 24 weeks of meldonium treatment in patients with peripheral arterial disease and intermittent claudication with meldonium [32]. In the second clinical trial, the improvement in exercise tolerance can be attributed to improved energy metabolism in skeletal muscles and their ability to perform better under partially ischemic conditions. In addition, as corroborated by some of the findings from the present study, exercise tolerance in patients with peripheral arterial disease was still improved one month after the discontinuation of meldonium therapy [32]. More detailed studies are needed to understand the exact site of action of meldonium in patients with RVF.

The SF-36 showed that patients had improved quality of life (QoL) [33]. QoL is a complex outcome that consists of an individual’s satisfaction in the physical, social, and psychological domains; unfortunately, an improvement in objective physical functioning does not always lead to an improvement in QoL [33,34,35]. An improvement over pretreatment SF-36 scores, especially in physical functioning, might be associated with increased exercise capacity [36]. There is still a debate among various conclusions regarding whether the QoL score can predict mortality or deterioration of disease; however, Blok et al. showed that in PAH CHD patients, a decrease in SF-36 PCS is a determinant of mortality; Mathai et al. showed that SF-36 scores are associated with survival in patients with PAH; and Johansson et al., in the Global Congestive Heart Failure Study, demonstrated that lower health-related QoL is associated with a higher risk of all unfavorable outcomes [37,38,39]. Jorge et al. compared QoL data among patients with and without heart failure, independent of the syndrome phenotype; they found significantly greater mean SF-36 scores in patients without HF than in those with HF, and the functional capacity of patients with HF was notably worse than that of patients without HF [40]. Therefore, we can hypothesize that treatment with meldonium positively influences SF-36 physical subscale scores toward those of the general population, which might mitigate HF patients’ increased risk for mortality due to primary disease.

In 2011, the SF-36 was used to determine health-related QoL in the Latvian population [41]. The physical functioning, role–physical, bodily pain, and general health parameters were significantly decreased in the RVF group in comparison to the population data. Treatment with meldonium increased SF-36 subscale scores as it increased exercise capacity, and RVF patients were able to perform their everyday duties. The emotional parameters did not differ greatly from those of the general population; however, 55% of the RVF patients were actively using psychopharmacological drugs that might improve their emotional well-being. After meldonium treatment, patients’ SF-36 scores even more closely approached those of the overall population.

Limitations of this observational study include its relatively short timeframe (limited to 60 days), particularly bearing in mind the chronicity of the disease, as well as the lack of a placebo control. Adverse reactions may occur in this severely ill patient population; however, since this is not a randomized controlled trial, it may be difficult to determine whether these adverse events are related to the relatively short duration of meldonium use or to other underlying factors. A longer follow-up time might be beneficial in further studies when the patients are randomized to the meldonium and they can be controlled and compared as well for adverse events. The observed findings in this study might be due to the composite effect of PAH-specific therapy and heart failure therapy received in addition to meldonium; on the other hand, the concomitant treatment was stable during the last 3 months with no augmentation of the functional parameters. The limitation is that the gold standard for assessing functional capacity remains the cardiopulmonary exercise test; therefore, the 6MWT provides only an estimate of it. Another limitation is the small sample size; however, the overall prevalence of the disease is low, and the patients, who were enrolled from the national PH registry, were representative of the average patient with RVF caused by PAH. Further clinical trials, including a placebo group, are needed to study the efficacy and safety of longer treatment periods, as well as to understand in more detail the mechanism and site of action of meldonium and its effects on RV function.

## 5. Conclusions

Meldonium treatment is safe and well tolerated, and it increases functional capacity and decreases dyspnea in patients with chronic RVF. Our results suggest that meldonium might be a viable novel drug treatment to improve the QoL of patients with RVF.

## Figures and Tables

**Table 1:** Clinical characteristics of the patients.

Characteristics	Overall Study Group; N = 20
Women, No. (%)	15 (75)
Age, mean ± SD, years	70.4 ± 13.2
BMI, kg/m^2^	28.1 ± 6.0
Time from PAH diagnosis, median (IQR) range, years	3.0 (0.9–4.0)
WHO FC II/III, (%)	35/65
**Comorbidity**	
Arterial hypertension, No. (%)	13 (65)
CHD, No. (%)	4 (20)
VTE in anamnesis, No. (%)	0 (0)
Diabetes mellitus, No. (%)	3 (15)
Thyroid dysfunction, No. (%)	2 (10)
OSA, No. (%)	1 (5)
Current smoking, No. (%)	1 (5)
**Medication**	
Anticoagulants, No. (%)	12 (60)
Digoxin, No. (%)	11 (55)
Spironolactone, No. (%)	18 (90)
Loop diuretics, No. (%)	18 (90)
Statins, No. (%)	14 (70)
ACEis, ARBs, or ARNIs, No. (%)	6 (30)
ACEis or ARBs plus thiazide, No. (%)	3 (15)
PDE5is, No. (%)	10 (50)
PDE5is plus ERAs, No. (%)	4 (20)
PDE5is, ERAs, and CCBs, No. (%)	3 (15)
PDE5is plus CCBs, No. (%)	1 (5)
ERAs plus CCBs, No. (%)	2 (10)
**Use of psychopharmacological drugs**	
Benzodiazepines, No. (%)	6 (30)
Antipsychotics, No. (%)	2 (10)
Hypnotics, No. (%)	3 (15)

ACEis—angiotensin-converting enzyme inhibitor, ARBs—angiotensin receptor blockers, ARNIs—angiotensin receptor–neprilysin inhibitor, BMI—body mass index, CCBs—calcium channel blockers, CHD—coronary heart disease, ERAs—endothelin receptor antagonists, OSA—obstructive sleep apnea, PAH—pulmonary arterial hypertension, PDE5is—phosphodiesterase type 5 inhibitors, VTE—venous thromboembolism, WHO FC—World Health Organization functional class.

**Table 2:** SF-36 subscale scores at the baseline visit, after 30 days and at the follow-up visit.

	Baseline Measurement, Points	Measurement After 30 Days, Points	Measurement at Follow-Up, Points
**Physical functioning**	58.0 ± 25.9	71.0 ± 23.0 *	67.8 ± 25.8 *
**Role limitations due to physical health**	60.0 ± 42.5	85.0 ± 28.6 *	73.8 ± 40.1
**Role limitations due to emotional problems**	90.0 ± 26.7	95.0 ± 16.3	88.3 ± 27.1
**Energy/fatigue**	76.5 ± 21.0	82.3 ± 15.2	76.0 ± 21.9
**Emotional well-being**	86.4 ± 15.9	86.2 ± 16.7	81.8 ± 17.1 *
**Social functioning**	86.9 ± 21.6	92.5 ± 19.6	88.1 ± 23.5
**Pain**	67.5 ± 30.6	83.3 ± 24.1 *	89.1 ± 16.7 *
**General health**	55.8 ± 23.2	61.3 ± 20.5	52.3 ± 23.6

The results are shown as the mean ± SD of 20 patients. * *p* < 0.05 vs. baseline measurement, Wilcoxon signed-rank test.

## Data Availability

The data reported in the article are stored in the Rare Disease Department of P. Stradins Clinical University Hospital, Riga, Latvia. The data underlying this article will be shared by the corresponding author on reasonable request.

## Abbreviations

The following abbreviations are used in this manuscript:

6MWT	six-minute walk test
ACEis	angiotensin-converting enzyme inhibitor
Aes	adverse events
ARBs	angiotensin receptor blockers
ARNIs	angiotensin receptor–neprilysin inhibitor
BDS	Borg dyspnea score
BMI	body mass index
CCBs	calcium channel blockers
CHD	coronary heart disease
CHF	chronic heart failure
CTD PAH	PAH associated with connective tissue disease
ERAs	endothelin receptor antagonists
IPAH	idiopathic PAH
LV	left ventricle
MCS	mental component summary
NYHA	New York Heart Association
OSA	obstructive sleep apnea
PAH	pulmonary arterial hypertension
PCS	physical component summary.
PDE5is	phosphodiesterase type 5 inhibitors
PH	pulmonary hypertension
QoL	quality of life
RHC	Right heart catheterization
RV	right ventricle
RVEF	RV ejection fraction
RVF	RV failure
SAEs	serious adverse events
SF-36	36-Item Short Form Survey
VTE	venous thromboembolism
WHO	World Health Organization
WHO FC	World Health Organization functional class
wmc	The weighted median change

## Appendix A

**Table A1:** The findings from RHC and transthoracic echocardiography of the patients.

Right Heart Catheterization	Measure ± SD
RAP, mmHg	8.2 ± 4.7
Mean pulmonary arterial pressure, mmHg	42.0 ± 13.4
Cardiac output, L/min	4.6 ± 1.1
Cardiac index, L/min/m^2^	2.2 ± 0.9
Pulmonary capillary wedge pressure, mmHg	12.4 ± 5.6
Pulmonary vascular resistance, Wood units	7.3 ± 5.1
Transthoracic echocardiography	
Aorta, mm	33.6 ± 4.5
LAVI, mL/m^2^	44.4 ± 18.2
IVSd, mm	11.0 ± 2.0
PWd, mm	10.3 ± 1.5
LVEF, %	60.0 ± 4.2
LVEDD, mm	49.0 ± 6.8
LVESD, mm	30.6 ± 7.0
LVMI, g/m^2^	107.2 ± 29.5
RVOT, mm	30.7 ± 2.1
RVD, mm	41.3 ± 8.3
RVSP, mmHg	66.8 ± 16.2
TAPSE, mm	20.3 ± 3.4
IVC, mm	18.7 ± 4.2
IVC collapse, >50%	N = 13
Pericardial effusion	N = 0

IVC—inferior vena cava; IVSd—interventricular septum at end-diastole; LAVI—left atrial volume index; LVEDD—left ventricular end-diastolic diameter; LVEF—left ventricular ejection fraction; LVESD—left ventricular end-systolic diameter; LVMI—left ventricular mass index; PWd—posterior wall thickness; RAP—right atrial pressure; RVD—right ventricular diameter; RVOT—right ventricular outflow tract; RVSP—right ventricular systolic pressure; SD—standard deviation; TAPSE—tricuspid annular plane systolic excursion.

**Table A2:** Vital parameters of the patients during the study period.

Parameter/Time Point	Baseline Measurement Before 6MWT	Baseline Measurement After 6MWT	Measurement Before 6MWT After 30 Days	Measurement After 6MWT After 30 Days	Measurement Before 6MWT After 60 Days	Measurement After 6MWT After 60 Days
**SBP, mmHg**	134.6 ± 15.3	141.7 ± 18.9	132.1 ± 19.8	139.1 ± 19.6	130.6 ± 16.9	137.9 ± 19.2
**DBP, mmHg**	79.4 ± 9.7	80.5 ± 10.8	79.9 ± 10.9	74.4 ± 12.7 *	77.1 ± 11.8	80.1 ± 10.0
**Heart rate, bpm**	71.4 ± 8.7	85.7 ± 13.9	76.2 ± 13.4	82.9 ± 14.8	72.9 ± 10.2	80.0 ± 14.3 *
**SpO2, %**	95.8 ± 2.2	92.2 ± 5.7	95.3 ± 3.9	93.6 ± 4.3	95.6 ± 2.3	94.2 ± 4.0
**RR, rpm**	17.9 ± 1.8	19.8 ± 3.5	18.1 ± 0.5	20.5 ± 3.3	17.9 ± 0.8	20.3 ± 2.1

The results are shown as the mean ± SD of 20 patients. * *p* < 0.05 vs. baseline measurement; the parametric *t* test was used for heart rate, SBP and DBP before and after the 6MWT at baseline, 30 days and 60 days, and the nonparametric Wilcoxon signed-rank test was used for SpO2 and RR at baseline, 30 days and 60 days. bpm—beats per minute; DBP—diastolic blood pressure; SBP—systolic blood pressure; rpm—respirations per minute; RR—respiration rate; SpO2—oxygen saturation; 6MWT—six-minute walk test.

**Table A3:** Results of the blood analysis of the patients before and after treatment with meldonium.

	Baseline Measurement	Measurement After 30 Days
**Erythrocytes, 10^12^/L**	4.5 ± 0.6	4.5 ± 0.6
**Hemoglobin, g/L**	135.0 ± 16.3	133.6 ± 14.3
**Platelets, 10^9^/L**	219 ± 50.4	224.6 ± 54.3
**Leucocytes, 10^9^/L**	7.1 ± 2.1	7.0 ± 1.7
**Neutrophils, 10^9^/L**	4.7 ± 1.7	4.7 ± 1.5
**Urea, mmol/L**	8.8 ± 2.9	8.8 ± 2.7
**Creatinine, µmol/L**	103.1 ± 29.7	103.5 ± 32.5
**Bilirubin, µmol/L**	13.4 ± 6.9	14.6 ± 5.7
**ALAT, U/L**	22.9 ± 13.0	22.9 ± 12.3
**ASAT, U/L**	28.7 ± 8.1	28.0 ± 8.1
**BNP, pg/ml**	159.0 ± 133.9	165.3 ± 135.9

The results are shown as the mean ± SD of 20 patients. ALAT—alanine transaminase; ASAT—aspartate transaminase; BNP—B-type natriuretic peptide.
